# Gender specific sexual dysfunction in patients with depression

**DOI:** 10.3389/fpsyt.2023.1194228

**Published:** 2023-06-15

**Authors:** Xinyu Liu, Zizhao Feng, Britta Galling, Na Qi, Xue-quan Zhu, Le Xiao, Gang Wang

**Affiliations:** ^1^The National Clinical Research Center for Mental Disorders and Beijing Key Laboratory of Mental Disorders, Beijing Anding Hospital, Capital Medical University, Beijing, China; ^2^Department of Child and Adolescent Psychiatry and Psychotherapy, Centre for Integrative Psychiatry, School of Medicine, Kiel, Germany; ^3^Department of Child and Adolescent Psychiatry, Psychosomatic Medicine and Psychotherapy, Charité-Universitätsmedizin Berlin, Berlin, Germany

**Keywords:** major depressive disorder, sexual dysfunction, gender, ASEX, female

## Abstract

**Background:**

This study aims to investigate the factors associated with sexual dysfunction (SD), with a particular focus on the influence of sex on the occurrence and severity of this condition in patients with major depressive disorder (MDD).

**Method:**

Sociodemographic and clinical assessments were conducted on 273 patients with MDD (female = 174, male = 99), including the ASEX, QIDS-SR16, GAD-7, and PHQ-15. Univariate analyses, independent samples *t*-test, Chi-square test, and Fisher’s exact test were used as appropriate, and logistic regression analysis was used to identify correlation factors for SD. Statistical analyses were performed using the Statistical Analysis System (SAS 9.4).

**Result:**

SD was reported in 61.9% of the participants (ASEX score = 19.6 ± 5.5), and the prevalence of it in females (75.3%, ASEX score = 21.1 ± 5.4) was significantly higher than that in males (38.4%, ASEX score = 17.1 ± 4.6). Factors associated with SD included being female, being aged 45 years or above, having a low monthly income (≤750 USD), feeling more sluggish than usual (a QIDS-SR16 Item 15 score of 1 or above), and having somatic symptoms (evaluated with the total score of PHQ15).

**Limitation:**

The use of antidepressants and antipsychotics might be a confounding factor affecting sexual function. Also, the lack of information in the clinical data regarding the number, duration, and time of onset of the episodes limits the richness of the results.

**Conclusion:**

Our findings reveal the sex differences in the prevalence and severity of SD in patients with MDD. Evaluated with the ASEX score, female patients showed significantly worse sexual function than male patients. Being female, having a low monthly income, being aged 45 years or above, feeling sluggish, and having somatic symptoms may increase the risk of SD in patients with MDD.

## 1. Introduction

The symptoms of major depressive disorder (MDD) include emotional, cognitive, and somatic symptoms (1, 2). In addition to the core symptoms, sexual dysfunction (SD) is reported by a majority of patients with MDD (3) and is associated with a low quality of life (4). Despite extensive research on MDD, SD has rarely been examined or considered as an associated factor of MDD. According to the Diagnostic and Statistical Manual of Mental Disorders, Fifth Edition (DSM-5™), SD is a clinically significant disturbance in a person’s ability to respond sexually or to experience sexual pleasure, including delayed ejaculation, erectile disorder, female orgasmic disorder, female sexual interest/arousal disorder, genitopelvic pain/penetration disorder, and others (5).

In the general population, the prevalence of SD in the United States is 43% for women and 31% for men (6). According to an epidemiological summary of sexual dysfunction in Asia, 37% of men and 45% of women in China have at least one type of sexual dysfunction (7). Although there are some regional variations in the reported prevalence of SD, most of the literature shows a higher prevalence of sexual dysfunction in women than in men (7, 8). The most common sexual dysfunctions in men are erectile dysfunction and premature ejaculation; in women, those are low sexual desire and impaired sexual arousal (9). Recently, it has been reported that the prevalence of SD in patients with MDD ranges from 46.66 to 90% (4, 8, 10), much higher than that in the general population, and SD might have a great impact on the quality of life and prognosis of patients.

The pathogenesis and related factors of SD are complicated. Some researchers have surmised that SD may be associated with the patient’s age, education level, physical health, emotional state, social and economic status, and cultural background (8, 11, 12). Moreover, the severity of MDD and the use of antidepressants have also been considered associated with the development of SD (13). Research has also found that SD occurred more frequently and was more severe in female patients with MDD than in male patients (8, 12), with symptoms including restricted sex drive and arousal, inability to reach orgasm, and decreased satisfaction from orgasm (14).

As sexual activity and functioning are sensitive topics in Asian cultural contexts, epidemiological studies published on sexual disorders in China are rare. Even fewer studies have taken patients with MDD as study subjects. Moreover, the impact of SD might be underestimated in Eastern cultures as patients with MDD may often be reluctant to talk about SD, even when they are in a hospital for the treatment of MDD. As there is limited evidence regarding the occurrence and severity of SD in patients with MDD, especially with regard to sex differences, this study aims to compare the sex differences in the aforementioned issue in a non-Western sample and, therefore, provide deeper insights into the biological and cultural pathogenesis.

## 2. Methods

### 2.1. Study design and participants

This study is a *post-hoc* data analysis of a Chinese nationwide naturalistic, prospective multi-center study conducted from May 2016 to October 2017 (15). It was approved by the Ethics Committee of Beijing Anding Hospital, Capital Medical University in 2016 and was approved by the ethics committees of all participating units (approval number: 2016-Science-Research-No.37). All patients participated in the survey voluntarily and signed informed consent forms. The primary study included patients aged 18 years or older diagnosed with major depressive disorder (MDD) according to the International Classification of Diseases, Tenth Revision (ICD-10) criteria and treated with at least one antidepressant for 8–12 weeks at any of the 16 participating psychiatric outpatient centers. Patients with comorbid bipolar disorder, schizophrenia, schizoaffective disorder, or other mental disorders associated with medical conditions or substance use were excluded.

### 2.2. Assessments and outcomes

Sociodemographic and clinical characteristics were collected at baseline. We assessed the severity of depression and anxiety using the 16-item Quick Inventory of Depressive Symptomatology-Self-Report (QIDS-SR16) and the Generalized Anxiety Disorder 7-Item (GAD-7) scale. The 15-item Patient Health Questionnaire (PHQ-15) was used to assess somatic symptoms. The Arizona Sexual Experience Scale (ASEX) was used to screen for sexual dysfunction (SD).

The QIDS-SR16 covers all the nine symptoms of MDD described in DSM-5. The items are measured on a 4-point scale ranging from 0 to 3, with a higher score indicating a higher severity of the measured symptom. The total score ranges from 0 to 27. The Chinese version of the scale has good reliability and validity (Cronbach’s *α* = 0.86) (16).

The GAD-7 scale consists of seven items. Each item is scored from 0 to 3 points, and the total score, as the sum of the scores of all items, ranges from 0 to 21 points. A higher score indicates a more severe symptom. The Chinese version of the GAD-7 scale has good psychometric properties (Cronbach’s *α* = 0.92) (17).

Different from the Patient Health Questionnaire-9 (PHQ-9), which focuses on depressive symptoms, the PHQ-15 measures somatic distress. It contains 15 items rated on a 3-point Likert scale ranging from 0 (not bothered at all) to 2 (bothered a lot). The Chinese version showed satisfactory internal consistency and test–retest reliability in a Hong Kong population (Cronbach’s *α* = 0.89) (18).

The ASEX is a five-item, easy-to-use rating scale for five domains of sexual function (sex drive, arousal, vaginal lubrication, ability to reach orgasm, and satisfaction from orgasm). The score of each item ranges from 1 to 6, and the total score ranges from 5 to 30. SD is defined as (i) a total ASEX score of 19 or above, (ii) any item with a score of 5 or above, or (iii) any three items with a score of 4 or above. The ASEX is a reliable, valid, and sensitive tool to measure SD in both males and females (19). The Chinese version of ASEX was used in this study because it has been shown to have good psychometric properties in Chinese patients with MDD (Cronbach’s *α* = 0.85) (20). All participants were instructed by a psychiatrist before completing the self-rating scale.

### 2.3. Statistical analysis

All statistical analyses were conducted using the Statistical Analysis System (SAS 9.4). In the univariate analyses, independent samples *t*-test, Chi-square test, and Fisher’s exact test were used as appropriate. Logistic regression analysis was performed to explore the correlation of SD with sex, age, years of education, monthly income, each item of QIDS-SR16, depressive symptoms (QIDS items), anxiety symptoms (GAD-7), somatic symptoms (PHQ-15), and antipsychotics co-treatment. The statistical significance was set at *p* < 0.05 (two-sided).

## 3. Results

### 3.1. Attrition bias

A total of 428 eligible patients were approached for the study. After excluding 155 patients who did not complete the ASEX questionnaire or indicated that it was “not applicable” due to sexual inactivity, 273 patients (174 females, 99 males) with a mean age of 41.2 ± 11.6 (range = 19–67) were included in the analysis. There were significant differences in marital status and antipsychotic co-treatment between included and excluded male patients, as well as in marriage status, antipsychotic co-treatment, age, and years of education between included and excluded female patients (see Table 1 for details).

**Table 1:** Demographic and clinical characteristics of all participants.

Male	Included (*N* = 99)	Excluded (*N* = 37)	*t*/*χ*^2^	*p*
Age	40.9 ± 11.7	45.9 ± 16.3	−1.73	0.0904
Marital status				**0.0202**
Married/partnered	74 (75.5%)	22 (59.5%)		
Divorced/separated	1 (1.0%)	3 (8.1%)		
Widowed	1 (1.0%)	3 (8.1%)		
Single	22 (22.5%)	9 (24.3%)		
Living				0.464
alone	8 (8.2%)	5 (13.5%)		
with family (parent, spouse, sibling)	83 (84.7%)	30 (81.1%)		
with friends	3 (3.1%)	2 (5.4%)		
other	4 (4.1%)	0 (0.0%)		
Years of education	13.1 ± 4.0	11.4 ± 4.1	1.78	0.078
Monthly average revenue, USD			2.58	0.4602
≤150	12 (12.1%)	7 (18.9%)		
150–750	47 (47.5%)	20 (54.1%)		
750–1,500	21 (21.2%)	6 (16.2%)		
≥1,500	19 (19.2%)	4 (10.8%)		
First depressive episode	62 (62.6%)	22 (59.5%)	0.11	0.7352
Duration of current episode	21.3 ± 16.2	21.1 ± 19.6	0.06	0.1433
Duration of antidepressant	9.6 ± 1.6	9.4 ± 1.6	0.5	0.6183
Antidepressant monotherapy	69 (69.7%)	22 (59.5%)	1.28	0.2588
Antipsychotic co-treatment	34 (34.3%)	20 (54.1%)	4.37	**0.0366**
QIDS-SR16	7.6 ± 4.5	8.6 ± 5.1	−1.14	0.2558
GAD-7	4.4 ± 4.0	5.0 ± 5.2	0.0071	0.9328
PHQ-15	5.6 ± 4.2	6.9 ± 4.7	−1.46	0.1463
Female	Include (*N* = 174)	Exclude (*N* = 118)	*t*/*χ*^2^	*p*
Age	41.4 ± 11.6	51.7 ± 16.4	−6.25	**<0.0001**
Marital status			22.11	**<0.0001**
Married/partnered	147 (84.5%)	74 (63.3%)		
Divorced/separated	2 (1.2%)	9 (7.7%)		
Widowed	3 (1.7%)	10 (8.6%)		
Single	22 (12.6%)	24 (20.5%)		
Living				0.0803
alone	7 (4.0%)	13 (11.1%)		
with family (parent, spouse, sibling)	158 (90.8%)	97 (82.9%)		
with friends	7 (4.0%)	4 (3.4%)		
other	2 (1.2%)	3 (2.6%)		
Years of education	12.3 ± 3.7	10.7 ± 4.8	3.04	**0.0027**
Monthly average revenue, USD			4.65	0.1993
≤150	48 (27.6%)	32 (27.6%)		
150–750	90 (51.7%)	69 (59.5%)		
750–1,500	30 (17.2%)	10 (8.6%)		
≥1,500	6 (3.5%)	5 (4.3%)		
First depressive episode	101 (58.1%)	76 (64.4%)	1.19	0.275
Duration of current episode	20.6 ± 14.7	21.8 ± 16.7	0.51	0.4714
Duration of antidepressant	9.8 ± 1.7	9.7 ± 1.6	0.22	0.8237
Antidepressant monotherapy	129 (74.1%)	89 (75.4%)	0.06	0.8042
Antipsychotic co-treatment	54 (31.0%)	54 (45.8%)	6.54	**0.0105**
QIDS-SR16	7.4 ± 4.7	6.5 ± 4.1	1.61	0.1084
GAD-7	4.0 ± 4.1	3.3 ± 3.9	3.64	0.0564
PHQ-15	6.5 ± 4.5	5.5 ± 4.1	2.38	0.1227

QIDS-SR16, the 16-item quick inventory of depressive symptomatology-self-report; GAD-7, generalized anxiety disorder 7-item; PHQ-15, the 15-item patient health questionnaire; The bold are *p*-values that are less than 0.05 (indicating statistically significant differences).

### 3.2. Description of overall sample

The majority of the sample was married or partnered, lived with family, had a monthly average revenue of 150–750 USD (low and middle income), and had a high-school-level education. Mono-antidepressant treatment was used in 72.5% of the sample. Patient and illness characteristics did not significantly differ except for monthly income, which was higher in males with MDD (*p* < 0.0001) (Table 2).

**Table 2:** Sample characteristics (overall, males and females).

	Total (*N* = 273)	Male (*N* = 99)	Female (*n* = 174)	*t*/*χ*^2^	*p*
Age	41.2 ± 11.6	40.9 ± 11.7	41.4 ± 11.6	−0.37	0.7100
Education, year	12.6 ± 3.8	13.1 ± 4.0	12.3 ± 3.7	1.84	0.0665
Current episode, week	20.9 ± 15.2	21.3 ± 16.2	20.6 ± 14.7	0.0864	0.7688
Symptom severity					
Depression (QIDS-SR total score)	7.5 ± 4.6	7.6 ± 4.5	7.4 ± 4.7	0.28	0.7799
Anxiety (GAD-7 total score)	4.2 ± 4.0	4.4 ± 4.0	4.0 ± 4.1	1.09	0.2975
Somatic symptoms (PHQ-15 total score)	6.2 ± 4.4	5.6 ± 4.2	6.5 ± 4.5	2.51	0.1132
Age				2.13	0.3455
18–30	60 (22.0%)	24 (24.2%)	36 (20.7%)		
30–45	115 (42.1%)	45 (45.5%)	70 (40.2%)		
≥45	98 (35.9%)	30 (30.3%)	68 (39.1%)		
Marital status				–	0.1626
Married/partnered	221 (81.3%)	74 (75.5%)	147 (84.5%)		
Divorced/separated	3 (1.1%)	1 (1.0%)	2 (1.2%)		
Widowed	4 (1.5%)	1 (1.0%)	3 (1.7%)		
Single	44 (16.2%)	22 (22.5%)	22 (12.6%)		
Living				–	0.1791
alone	15 (5.5%)	8 (8.2%)	7 (4.0%)		
with family (parent, spouse, sibling)	241 (88.6%)	83 (84.7%)	158 (90.8%)		
with friends	10 (3.7%)	3 (3.1%)	7 (4.0%)		
other	6 (2.2%)	4 (4.1%)	2 (1.2%)		
Monthly average revenue, USD				24.71	**<0.0001**
≤150	60 (22.0%)	12 (12.1%)	48 (27.6%)		
150–750	137 (50.2%)	47 (47.5%)	90 (51.7%)		
750–1,500	51 (18.7%)	21 (21.2%)	30 (17.2%)		
≥1,500	25 (9.2%)	19 (19.2%)	6 (3.5%)		
Antidepressant treatment					
Antidepressant monotherapy	198 (72.5%)	69 (69.7%)	129 (74.1%)	0.62	0.4294
Antidepressant polypharmacy (≥2 antidepressants)	75 (27.5%)	30 (30.3%)	45 (25.9%)	0.62	0.4294
Antipsychotic treatment					
Antipsychotic monotherapy without antidepressant					
Antipsychotic-antidepressant co-treatment					
Antipsychotics co-treatment	88 (32.2%)	34 (34.3%)	54 (31.0%)	0.32	0.5739

n, number of patients; QIDS-SR16, the 16-item quick inventory of depressive symptomatology-self-report; GAD-7, generalized anxiety disorder 7-item; PHQ-15, the 15-item patient health questionnaire; The bold are *p*-values that are less than 0.05 (indicating statistically significant differences).

### 3.3. Sexual dysfunction in males and females with MDD

Of the 273 patients, 169 (61.9%) reported sexual dysfunction with a mean ASEX score of 19.6 ± 5.5. Females had a significantly higher frequency of sexual dysfunction than males (75.3% vs. 38.4%, *p* < 0.001), with low sex drive reported in 44% of all female patients and difficulty in sexual arousal reported in 26.7% of them. Among male patients, 11.1% had difficulty in penile erection; among female patients, 25.9% had difficulty in vaginal lubrication. Difficulty in reaching orgasm was reported in 29.7% of all patients, and unsatisfying orgasm was reported in 28.9% of them. Evaluated with the item scores and the total score of the ASEX, female patients experienced much more severe dysfunction in all sexual domains compared to male patients (all *p* < 0.01) (Table 3).

**Table 3:** Sexual dysfunction (overall*, males and females).

	Total *N* = 273	Male *N* = 99	Female *N* = 174	*P*
ASEX total score	19.6 ± 5.5	17.1 ± 4.6	21.1 ± 5.4	<0.0001
Positive item (≥5), *n* (%)				
quantify sex drive	120 (44.0%)	30 (30.3%)	90 (51.7%)	0.0006
arousal	73 (26.7%)	10 (10.1%)	63 (36.25%)	<0.0001
vaginal lubrication or penile erection	56 (20.5%)	11 (11.1%)	45 (25.9%)	0.0037
ability to reach orgasm	81 (29.7%)	16 (16.2%)	65 (37.4%)	0.0002
satisfaction from orgasm	79 (28.9%)	17 (17.2%)	62 (35.6%)	0.0012
Number of ASEX positive item	1.5 ± 1.9	0.8 ± 1.5	1.9 ± 2.0	<0.0001
Sexual dysfunction	169 (61.9%)	38 (38.4%)	131 (75.3%)	<0.0001

ASEX, Arizona Sexual Experience Scale; *N*, number of patients.

^*^Overall Sexual dysfunction was defined as (i) a total score ≥ 19, (ii) ≥ 5 in any item, or (iii) ≥ 4 in three items.

### 3.4. Factors associated with sexual dysfunction in patients with MDD

The logistic regression model for the overall sample showed that being female (OR = 4.5, 95%CI: 2.46, 8.24), being aged 45 years or above (OR = 2.33, 95%CI: 1.06, 5.10), low monthly income (≤750 USD, OR = 3.64, 95%CI: 1.91, 6.65), feeling more sluggish than usual (QIDS-SR16 Item 15 score ≥ 1, OR = 2.05, 95%CI: 1.11, 3.79), and having somatic symptoms (total score of PHQ15, OR = 1.09, 95%CI: 1.01, 1.17) were associated with sexual dysfunction (see Table 4).

**Table 4:** Factors associated with sexual dysfunction.

	*β*	SE	*χ* ^2^	*P*	OR (95% CI)
Age strata					
40–45 vs. 18–30	−0.31	0.20	2.38	0.1229	0.95 (0.46,2.00)
>45 vs. 18–30	0.58	0.22	7.12	0.0076	2.33 (1.06,5.10)
Female	1.50	0.31	23.85	<0.0001	4.50 (2.46,8.24)
Total score of PHQ15	0.08	0.038	4.65	0.0311	1.09 (1.01,1.17)
Income level*: ≤ 750 vs. >750	0.65	0.16	15.42	<0.0001	3.64 (1.91,6.95)
QIDS Item 15 (feeling more sluggish than usual) ≥ 1	0.36	0.16	5.32	0.0211	2.05 (1.11,3.79)

*USD; n, number of patients; QIDS, the 16-item quick inventory of depressive symptomatology-self-report; PHQ-15, the 15-item patient health questionnaire.

## 4. Discussion

We observed a significant sex difference in sexual dysfunction among patients with MDD. The prevalence of sexual dysfunction was significantly higher in female patients than in male patients. Female patients demonstrated significantly more severe dysfunction in all sexual domains, as indicated by both item and total scores of ASEX, than male patients. Our study suggests that sexual dysfunction in patients with MDD is likely associated with female sex, age above 45, low monthly income (≤ 750 USD), feeling more sluggish than usual (QIDS item 15 ≥ 1), and having somatic symptoms (evaluated with the total score of PHQ15).

An epidemiological summary of sexual dysfunction in Asia by Lewis et al. (7) reported that 37% of men and 45% of women in China have at least one type of sexual dysfunction. In the general population, women typically report more sexual functioning problems than men (21). The prevalence of sexual dysfunction is much higher in MDD patients than in the general population (8, 10, 22). A meta-analysis of 12 cross-sectional studies on depressive and persistent depressive disorders revealed that 82.75% of women (95% CI: 74.71–90.78) and 63.26% of men (95% CI: 52.83–73.69) experienced general sexual dysfunction (23). Our study found that, according to self-assessment results, 61.9% of patients with MDD experienced sexual dysfunction, with a significantly higher prevalence of sexual dysfunction in females (75.3%) than in males (38.4%). Our results are consistent with previous reports on the prevalence of sexual dysfunction, indicating a higher prevalence of sexual dysfunction in females with MDD than in males with MDD. Sex differences in sexual dysfunction in patients with MDD may be attributed to underlying neurobiological mechanisms (24). Patients with MDD exhibit decreased activation in various brain regions during visually evoked sexual arousal: males show decreased activation in the hypothalamus, thalamus, caudate nucleus, and temporal gyrus, and females show decreased activation in the para-hippocampal gyrus and anterior cingulate cortex, which are vulnerable during MDD episodes (25, 26). Difficulty in communicating with life partners or the influences of East Asian cultures on the patients may also contribute to these results. The prevalence of sexual dysfunction in patients with MDD is associated with the severity of depression (27). Our results indicate that the severity of depression was similar between the male and female groups (there was no significant difference in the QIDS-SR total score between the male and female groups); therefore, the effect of sex on the prevalence of sexual dysfunction in patients with MDD was not substantially affected by the severity of depression.

In our study, female patients had higher scores across all domains of sexual dysfunction in the ASEX, including low sex drive, difficulty with sexual arousal, difficulty in achieving penile erection or vaginal lubrication, difficulty in achieving orgasm, and unsatisfying orgasm. Lai et al. ((14)) reported similar findings, with females (the mean ASEX score was 20.88) exhibiting significantly higher ASEX scores than males (the mean ASEX score was 16.93). Although some studies have suggested that ASEX scores are associated with the severity of depressive symptoms (10, 14), we found no significant correlation between the QIDS-SR16 total score and the ASEX score in our study. However, the occurrence of SD was indeed associated with one item of the QIDS-SR16 (the score of Item 15 feeling more sluggish than usual ≥1). In a 6-month follow-up study, we found no significant difference between the ASEX total scores of patients with or without remission, and sexual dysfunction did not improve with the alleviation of depressive symptoms (15). Therefore, our results suggest that sexual dysfunction in patients with MDD may be relatively independent of the severity of depressive symptoms, but may be affected by certain domains of MDD symptoms such as feeling sluggish. Nonetheless, it should be noted that some studies have suggested a strong association between SD and depressive symptoms evaluated with objective scales such as the HDRS (14, 28) or MADRS (29). The difference in evaluation tools may account for the varied conclusions.

Our results reveal that being female and feeling more sluggish than usual (a QIDS-SR16 Item 15 score ≥ 1) are associated with SD, along with being aged 45 years or above, having a low monthly income, and having somatic symptoms (evaluated with the total score of PHQ15). These factors may predict the likelihood of developing SD in patients with MDD, and we will discuss each factor individually below.

The mean age of our study population was 41 years for both male and female groups. Previous research suggests that sexual dysfunction may increase with age (3, 30). As women age, they experience a reduction in pelvic muscle tension, relaxation of the urethral meatus, a decrease in orgiastic rectal contractions, vaginal dryness associated with declining estrogen, and reduced libido (possibly linked to a decline in androgen levels (31)). In males, age-related changes in sexual response include a decrease in libido, the number and frequency of morning erections, penile sensitivity, and arousal, as well as a longer time needed to achieve and maintain an erection, prolonged plateau phase, reduced ejaculatory volume and force of expulsion, and prolonged refractory period (32). Being aged 45 years or above is a risk factor for sexual dysfunction in both male and female patients with MDD.

Low monthly income (≤ 750 USD) may result in high pressure regarding family responsibilities and a depressed sexual environment, leading to dissatisfaction with sexual function among patients, which could explain part of our results. Additionally, our study found that compared to men, a significantly higher proportion of women had low income, which may contribute to the high incidence of SD in women. Kim et al. (33) also suggested a correlation between female sexual dysfunction and lower economic income; moreover, lower socioeconomic status (measured by the poverty income ratio) and lower sexual frequency are directly correlated among female adults in the United States. A cross-sectional study on rural Iranian women found that financial stress and lack of support increased the likelihood of depression and the risk of sexual dysfunction (11).

One meta-analysis showed that poor physical condition was a significant predictor of SD (34), which is in line with our findings. A study on women in Hong Kong, China, also found that moderate or poor self-rated physical health, measured with a single item (“How would you evaluate your health status?”) on a five-point Likert scale (ranging from “1 = very good” to “5 = very poor”), was associated with sexual dysfunction (35). There is a close correlation between physical discomfort and sexual dysfunction both in patients with MDD and the general population. It is worth noting that our study found that the total score of GAD-7 (indicating the severity of anxiety) was not a contributing factor to sexual dysfunction, which is consistent with some previous findings (4, 12).

This study has several limitations that should be considered. Firstly, we did not take into account the influence of attitudes toward sex activities and the mastery of sex-related knowledge, which have been shown to significantly affect the occurrence of sexual dysfunction (8). Secondly, the use of antidepressants and antipsychotics may affect sexual function (36), potentially contributing to the prevalence of sexual dysfunction in our results. However, as there was no significant difference in the use of these medications between the male and female groups, the possible impact of these confounding factors can be excluded. Thirdly, more detailed clinical information regarding the onset, number of episodes, and duration of sexual dysfunction would have strengthened the analysis and increased the generalizability of the results. Finally, the sample size was not calculated for the purpose of this study.

## 5. Conclusion

Our findings mainly revealed that there were sex differences in the prevalence and severity of SD in patients with MDD. Evaluated with the ASEX score, the sexual function of female patients was significantly worse than that of male patients. Being female, having a low monthly income, being older than 45 years old, feeling sluggish, and having somatic symptoms may increase the risk of SD in patients with MDD.

## Data availability statement

The original contributions presented in the study are included in the article/supplementary materials, further inquiries can be directed to the corresponding author.

## Ethics statement

The studies involving human participants were reviewed and approved by the Ethics Committee of Beijing An Ding Hospital, Capital Medical University. The patients/participants provided their written informed consent to participate in this study.

## Author contributions

LX: conceptualization, methodology, resources. XL: data curation, formal analysis, writing-Original draft preparation.ZF: data curation, writing-revised draft preparation, language polishing and editing. BG: supervision, data curation, project administration. NQ: data curation, formal analysis. XZ: methodology, data curation, software. GW: comments on first draft revisions, project administration. All authors listed have made a substantial, direct, and intellectual contribution to the work and approved it for publication.

## Funding

GW receives grant from Beijing Scholar 2021(No.063).

## Conflict of interest

The authors declare that the research was conducted in the absence of any commercial or financial relationships that could be construed as a potential conflict of interest.

## Publisher’s note

All claims expressed in this article are solely those of the authors and do not necessarily represent those of their affiliated organizations, or those of the publisher, the editors and the reviewers. Any product that may be evaluated in this article, or claim that may be made by its manufacturer, is not guaranteed or endorsed by the publisher.

## Abbreviations

QIDS-SR16: The 16-item quick inventory of depressive symptomatology-self-report
GAD-7: Generalized anxiety disorder 7-item
PHQ-15: The 15-item patient health questionnaire
MDD: Major depressive disorder
SD: Sexual dysfunction
ASEX: Arizona Sexual Experience Scale
